# Hyperbaric Oxygen Therapy Attenuates Burn-Induced Denervated Muscle Atrophy

**DOI:** 10.7150/ijms.65976

**Published:** 2021-10-25

**Authors:** Chin-An Chen, Yi-Chen Huang, Jing-Jou Lo, Shih-Hung Wang, Shu-Hung Huang, Sheng-Hua Wu

**Affiliations:** 1 Department of Anesthesiology, Kaohsiung Municipal Ta-Tung Hospital, Kaohsiung, Taiwan.; 2 Department of Anesthesiology, Kaohsiung Medical University Hospital, Kaohsiung, Kaohsiung Medical University, Taiwan.; 3 School of Medicine, College of Medicine, Kaohsiung Medical University, Kaohsiung, Taiwan.; 4 Department of General Medicine, Kaohsiung Medical University Hospital, Kaohsiung Medical University, Kaohsiung, Taiwan.; 5 Division of Plastic Surgery, Department of Surgery, Kaohsiung Medical University Hospital, Kaohsiung Medical University, Kaohsiung, Taiwan.; 6 Department of Surgery, School of Medicine, College of Medicine, Kaohsiung Medical University, Kaohsiung, Taiwan.; 7 Hyperbaric Oxygen Therapy Center, Kaohsiung Medical University Hospital, Kaohsiung Medical University, Kaohsiung, Taiwan.; 8 Department of Anesthesiology, School of Medicine, College of Medicine, Kaohsiung Medical University, Kaohsiung, Taiwan.

**Keywords:** Burn injury, Hyperbaric oxygen therapy, Neuronal apoptosis, Denervated muscle atrophy, Hypoxia-inducible factor-1α

## Abstract

**Background:** Neuronal apoptosis and inflammation in the ventral horn of the spinal cord contribute to denervated muscle atrophy post-burn. Hyperbaric oxygen therapy (HBOT) exerts anti-inflammation and neuroprotection. Furthermore, hypoxia-inducible factor (HIF)-1α has been reported to promote inflammation and apoptosis. We investigated the therapeutic potential of HBOT and the role of HIF-1α post-burn.

**Methods:** Sprague-Dawley rats were divided into three groups: a control group, an untreated burn group receiving burn and sham treatment, and a HBOT group receiving burn injury and HBOT. The burn injury was induced with 75ºC ± 5ºC at the right hindpaw. HBOT (100% oxygen at 2.5 atmosphere, 90 min/day) and sham HBOT (21% oxygen at 1 atmosphere, 90 min/day) was started on day 28 after burn injury and continued for 14 treatments (days 28-41). Incapacitance (hind limb weight bearing) testing was conducted before burn and weekly after burn. At day 42 post-burn, the gastrocnemius muscle and the spinal cord ventral horn were analyzed.

**Results:** HBOT improved burn-induced weight bearing imbalance. At day 42 post-burn, less gastrocnemius muscle atrophy and fibrosis were noted in the HBOT group than in the untreated burn group. In the ventral horn, HBOT attenuated the neuronal apoptosis and glial activation post-burn. The increases in phosphorylated AKT/mTOR post-burn were reduced after HBOT. HBOT also inhibited HIF-1α signaling, as determined by immunofluorescence and western blot.

**Conclusions:** HBOT reduces burn-induced neuronal apoptosis in the ventral horn, possibly through HIF-1α signaling.

## Introduction

Skeletal muscle wasting is a common features after burn injury and may last for months to years 1-5. It is often associated with several complications and impaired rehabilitation. However, limited research has investigated the underlying molecular mechanism, and an effective strategy to attenuate the long-term muscle atrophy after burn injury is lacking. Burns can induce excessive protein degradation in muscle 6, and our previous study suggested that neuronal apoptosis in the spinal cord ventral horn contributes to denervated gastrocnemius muscle atrophy 7. Neuroinflammation after burn injury also increases distant muscle mass loss 8.

Hyperbaric oxygen therapy (HBOT) involves patients breathing 100% oxygen at between 1.5 and 3.0 standard atmospheres for an average duration of 90 mins. It is generally employed to treat conditions such as decompression sickness, carbon monoxide poisoning, and problem wounds 9. HBOT attenuates inflammation and oxidative reactions by improving tissue oxygen starvation in experimental-induced skeletal muscle injury and colitis models 10, 11. Moreover, the neuroprotective effects of HBOT reduce cognitive impairment 12-14. Mu *et al* reported that delayed HBOT (started 48 h after ischemic brain injury) increased regenerative cell proliferation 15. Data increasingly support the benefits of HBOT, including neuronal apoptosis inhibition, neuroinflammation reduction, and anti-oxidative capacity 16-20. Dave *et al.* indicated that HBOT protected against mitochondrial dysfunction in a mouse model of motor neuron disease 21. Based on these evidence, we supposed a potential therapeutic effect of HBOT for attenuating neuronal apoptosis and inflammation in the ventral horn of the spinal cord after burn injury.

Previous research showed that HBOT improved wound healing by inhibiting hypoxia-inducible factor-1α (HIF-1α) overexpression in an ischemic wound model 22. Nakazawa *et al.* reported that burn-induced mitochondrial dysfunction and activation of the HIF-1α pathway in mouse skeletal muscle 23. The HIF-1α pathway also plays critical roles in several central nervous system (CNS) disorders; however, the function of HIF-1α in CNS impairments remains controversial. Upregulated HIF-1α expression has promoted programmed neuronal death in rat models of traumatic brain injury 24, 25, cerebral ischemia 26, 27, and spinal cord injury 28. Furthermore, inflammatory stimuli are potent activators of HIFs under normoxic conditions and targeting HIF activity is a potential strategy for tissue repair in inflammatory disorders 29-31. The HIF-1α signaling axis is also associated with the activation of abnormal glial inflammation and neuronal cell death in during cerebral ischemia 32. Guo *et al.* indicated that HIF-1α axis activation enhanced mesenchymalstromal cells migration and reduced neuronal apoptosis in traumatic brain injury 33. Methods to upregulate the HIF-1α signaling pathway 34, 35 and the administration of recombinant adenovirus expressing HIF-1α 36 have also been proposed to attenuate neuronal apoptosis in cerebral ischemic injury. In the present study, we investigates whether HBOT exerts neuroprotective effects by targeting HIF-1α signaling in a burn-induced denervated muscle atrophy rat model.

## Materials and Methods

### Animals and Experimental Design

**Figure 1:**
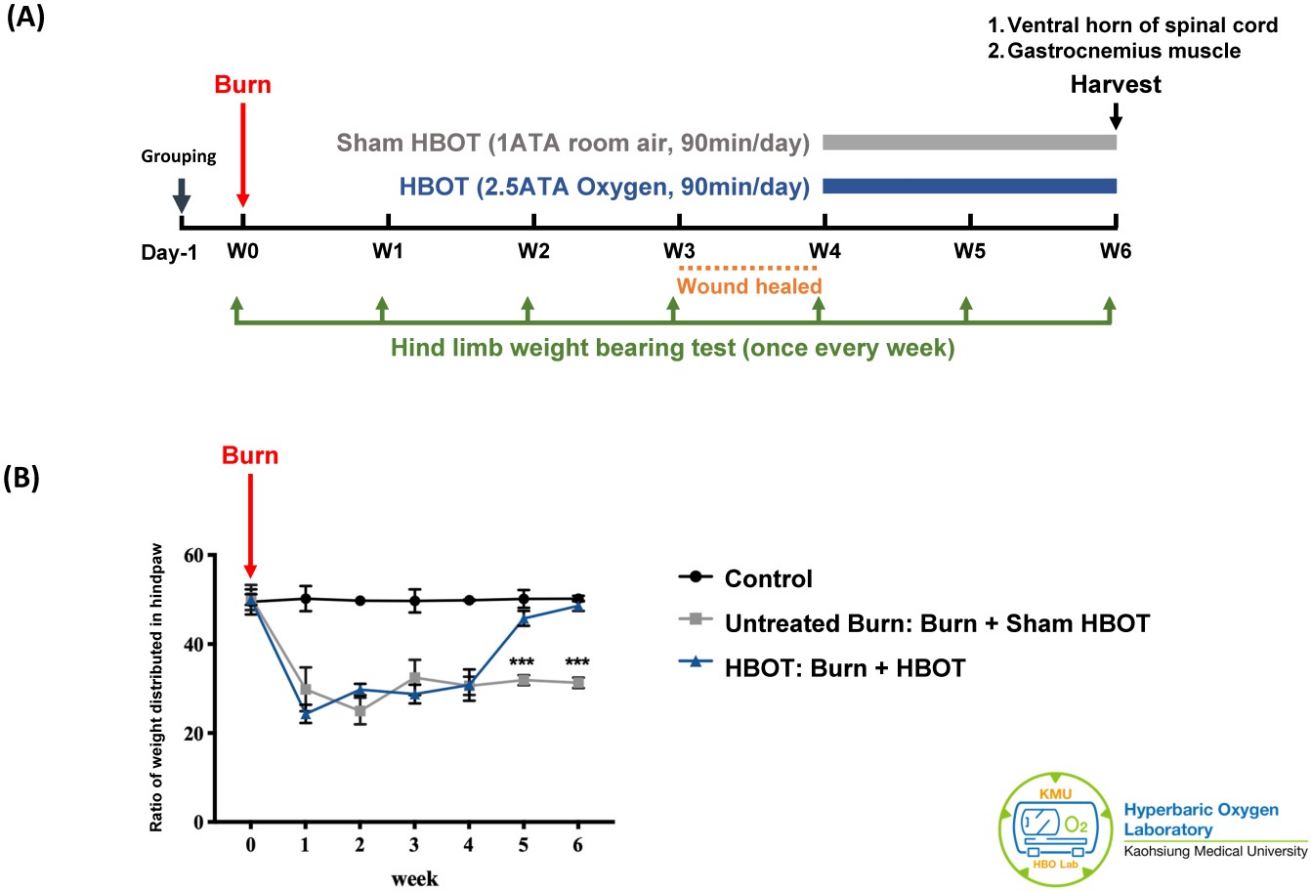
** Experimental design and behavior test results. (A)** Time course of experimental design. **(B)** Incapacitance test results of each group at indicated time points. Ratio of weight distributed between an injured and non-injured hindpaw was measured. HBOT significantly improved the weight bearing of right hindpaw on week 5 and week 6 post-burn. Data are displayed as mean ± standard deviation. ***p < 0.001 (n=6 rats per group).

The experiment was approved by the Institutional Animal care and Use Committee of Kaohsiung Medical University (IACUC Approval Number: 109045). A total of 18 adult male Sprague-Dawley rats (weighting 150-175 g) (BioLASCO Taiwan Co., Ltd) were randomly allocated into three groups, with 6 rats in each group. According to previous researches, the group size is generally adequate for detection of significant biological effects in animals 37, 38. The control group received a sham burn injury and sham treatment. The untreated burn group received a burn injury and sham HBOT. The HBOT group received a burn injury and daily HBOT for 2 weeks. Figure 1A presents the timeline of experimental design. Burn injury was induced on day 0 as described previously 7. The wounds were cared with silver sulfadiazine ointment daily until they healed, approximately 3-4 weeks after injury. The rats in the HBOT group were placed in a hyperbaric chamber (Genmall Biotechnology Co., Ltd., Taiwan) and received HBOT (100% oxygen at 2.5 ATA for 90 mins) 39, 40 on days 28 to 41 after burn. The rats in the other two groups were placed inside the same chamber and received room air (21% oxygen at 1 atmosphere for 90 min/day) as a sham treatment. An incapacitance meter (Singa Technology, Taipei, Taiwan) was used to measure the ratio of weight distributed between an injured and non-injured hindpaw, while normal rats distribute weight 50-50. Measurement was performed on day 0 before burn injury and once weekly after burn until the rats were be sacrificed. The weight balance tests were performed over a 5-s period for 3 measurements, and the change in hindpaw weight distribution was calculated as described previously 41.

At 6 weeks after burn injury, the rats were anesthetized with the use of Zoletil 50 (50 μg/g; Virbac Laboratory, Australia). The gastrocnemius muscle and ventral horn of the spinal cord (L3-5 segments) were harvested. Gastrocnemius tissue sections were stained with hematoxylin and eosin (H&E), picrosirius red, and Masson's trichrome stains according to the manufacturers' instructions and visualized through light microscopy. The average muscle cross-sectional area of each group was acquired from six stained sections of each specimen by a Nikon Eclipse E600 microscope, and images were captured with a Nikon Digital Sight DS-5M imaging system. The Masson's trichrome-stained images was employed for fibrotic analysis with Image Pro-Plus 6.0 image analysis software (Media Cybernetics, Bethesda, MD, USA) after observation under a microscope. The muscle sections were also incubated overnight with muscle ring finger-1 (MuRF-1, 1:200; Bioss Antibodies, Beijing, China), and laminin (1:200; GeneTex, Irvine, CA, USA) for immunofluorescence analysis. Western blot of atrogin-1 (1: 1000; Affinity Biosciences, Changzhou, China) was performed to observe muscle atrophy, and caspase cascades (caspase-3, 1: 1000; Cell Signaling Technology, Beverly, MA, USA; caspase-9, 1: 1000; Novus Biologicals, Littleton, CO, USA) were evaluated in muscle sections. The protein bands were visualized using the ECL Western Blotting Detection Kit and Bio-Rad ChemiDoc XRS system. The band intensity was also quantified and plotted by Quantity One Software.

Furthermore, neuronal apoptosis in the ventral horn of the spinal cord was investigated with terminal deoxynucleotidyl transferase dUTP nick-end labeling (TUNEL) assay, and images were recorded with an inverted microscope (Leica DMI6000). Immunofluorescence staining for phosphorylated NFκB (p-NFκB, 1: 200; Cell Signaling Technology), glial fibrillary acidic protein (GFAP, 1: 500;Arigobio, Taiwan), cleaved caspase 3 (1: 1000; Cell Signaling Technology), HIF-1α (1:200; Bioss Antibodies, Beijing, China) and neuron-specific nuclear protein (NeuN, 1:1000; Millipore, Temecula, CA, USA) in the ventral horn was performed and analyzed through a fluorescence microscope (Leica DMI6000). In addition, Western blot was used to investigate p-NFκB (1: 1000; Cell Signaling Technology), p-IκB (1: 1000; Cell Signaling Technology), cleaved caspase 3 (1: 1000; Cell Signaling Technology), cleaved caspase 9 (1: 1000; Novus Biologicals), BCL-2-associated X (BAX, 1:1000; ProteinTech Group, Chicago, IL, USA), B-cell lymphoma 2 (BCL-2, 1:500; Abcam, Cambridge, MA, USA), phosphorylated AKT (p-AKT, 1:1000; Cell Signaling Technology), AKT (1:1000; Cell Signaling Technology), phosphorylated mammalian target of rapamycin (p-mTOR, 1:1000; Cell Signaling, Technology), mTOR (1:1000, Cell Signaling Technology), HIF-1α (1:500; Bioss Antibodies) and β-actin (1:5000; Novus Biologicals) in the ventral horn.

The significance of differences between groups was identified by paired Student's t-test or one-way ANOVA followed by the post hoc Dunnett's test for multiple comparisons as appropriate. All data were expressed as mean ± standard deviations (SD) and 95% confidence interval (CI), which are respectively indicated by bar graphs and error bars. SPSS version 14.0 (SPSS, Inc., Chicago, IL, USA) was used to analyze the experimental results. A p-value <0.05 was considered significant.

## Results

As Figure 1B shows, the weight-bearing capacity of right hindpaw was markedly decreased in the untreated burn group and lasted for 6 weeks after burn. The HBOT group exhibited greater weight-bearing capacity at days 35 and day 42 post-burn than did the untreated burn group (95% CI: 10.67-17.11, p=0.0003; 95% CI: 14.65-19.96, p=0.0001, respectively). These results indicate that HBOT improved the force distribution evenly.

**Figure 2:**
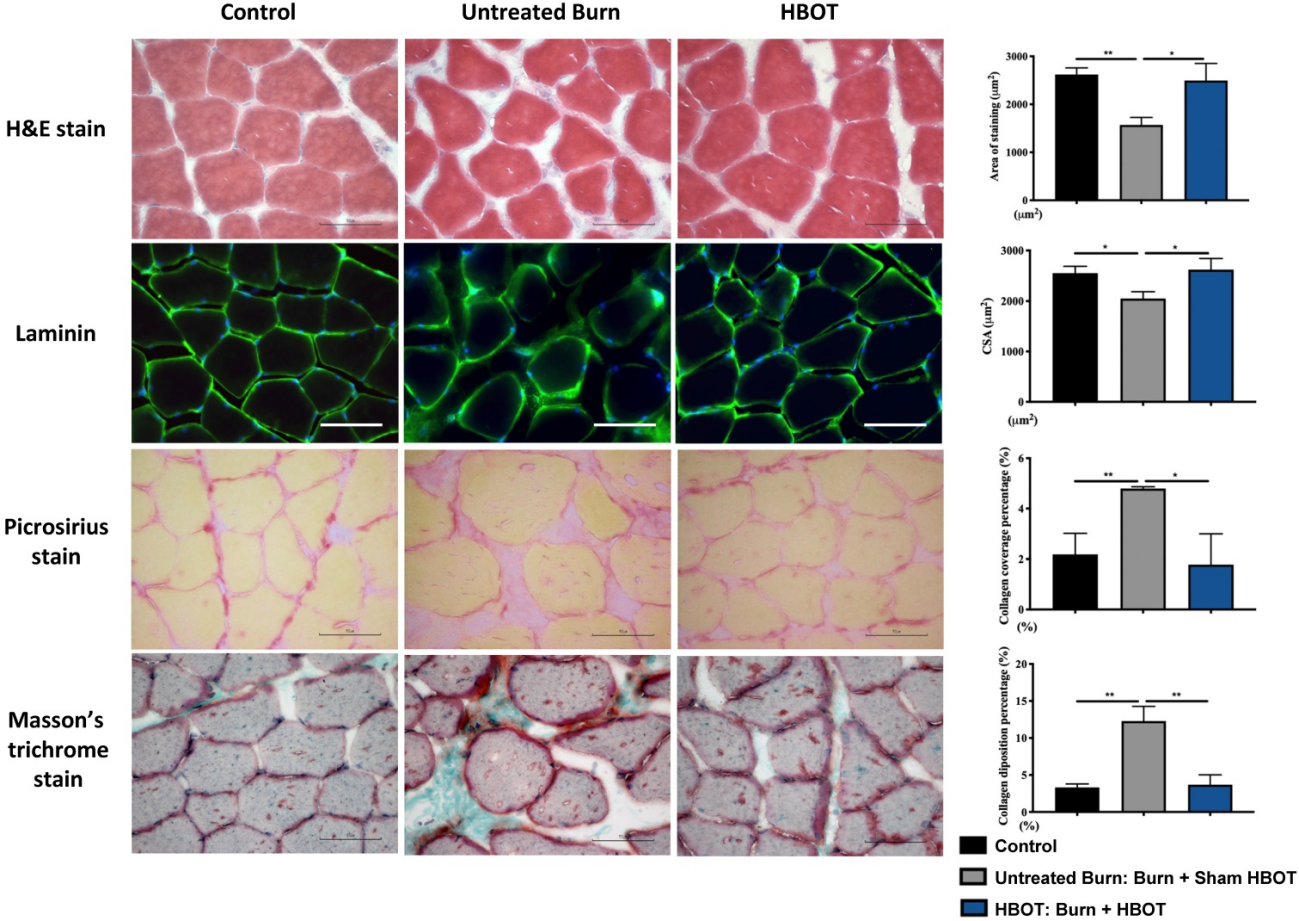
** HBOT improves burn-induced muscle atrophy and fibrosis by histological examination. (A)** Hematoxylin and Eosin (H&E) stain and **(B)** immunofluorescences of Laminin of gastrocnemius muscle cross-sections for each group at days 42 post-burn. HBOT improves the decrease of myofiber cross-sectional area post-burn. **(C)** Picrosirius red and **(D)** Masson's trichrome stain of gastrocnemius muscle section to display total collagen content. Results are shown as percentage area of collagen in gastrocnemius muscle section. There is a significant increase of collagen deposition in gastrocnemius muscle in untreated burn groups compared with control and HBOT groups. Original magnifications×400. Scale bars, 50 µm. *p < 0.05, **p < 0.01 compared with indicated group.

**Figure 3:**
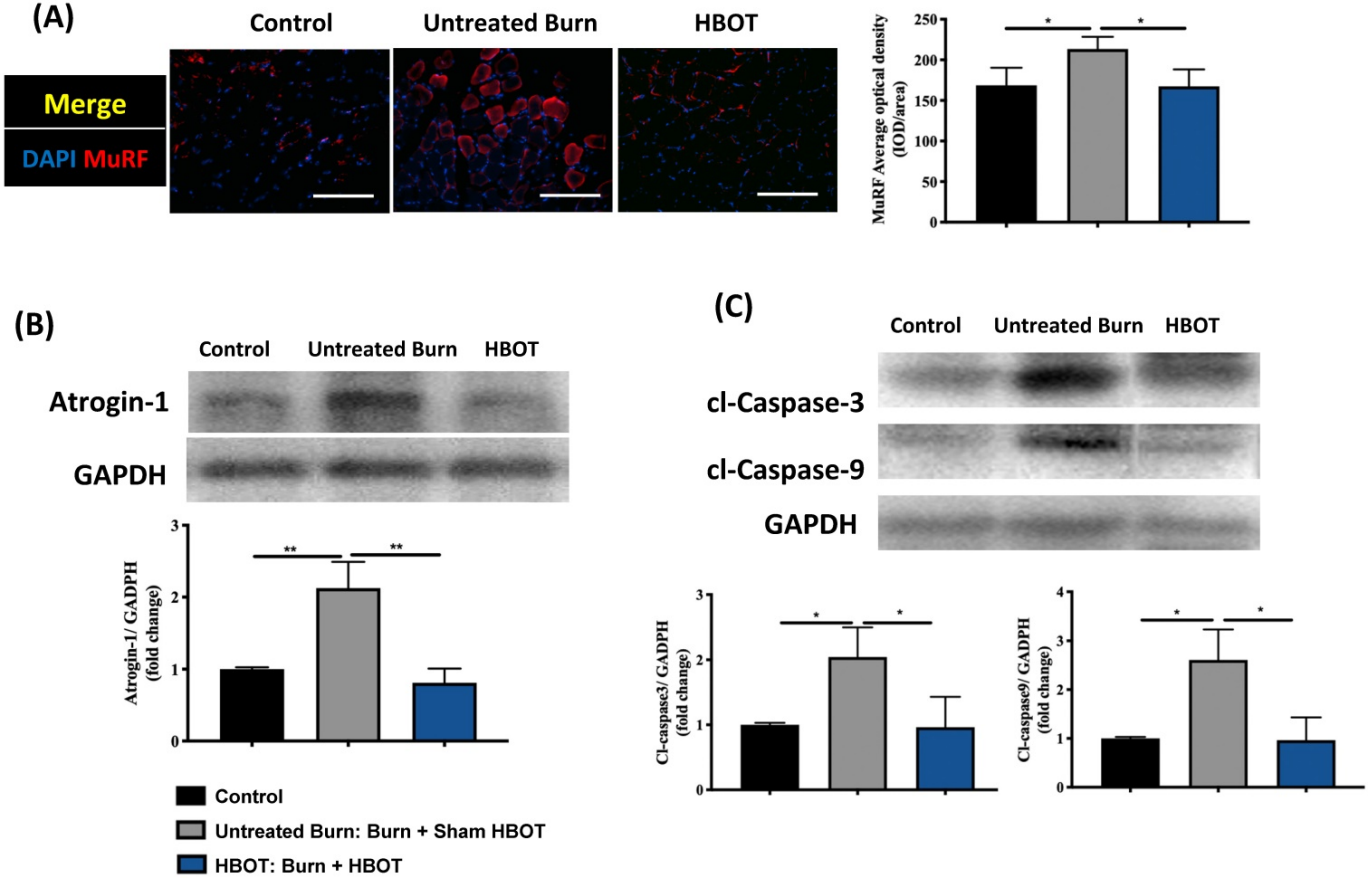
** HBOT attenuates burn-induced muscle wasting by immunofluorescence and western blot. (A)** Immunofluorescence of muscle ring finger-1 (MuF-1) (red) in gastrocnemius muscle. MuF-1 which participates in skeletal muscle atrophy, was up-regulated in untreated burn group and HBOT attenuated the phenomenon. Nuclei of the cells in the muscle sections were counterstained with DAPI (blue). **(B)** Western blot analysis of atrogin-1, an important regulators of ubiquitin-mediated protein degradation in skeletal muscle. HBOT attenuated a significant decrease of atrogin-1 following burn. **(C)** Western blot analysis of cl-caspase-3 and -9. HBOT also improve the up-regulation of caspase cascades post-burn. Scale bars, 50 µm. *p < 0.05, **p < 0.01 compared with indicated group.

Gastrocnemius muscle sections were stained with H&E and an anti-laminin antibody to investigate the muscle fiber architecture and cross-sectional area in Figure 2; burn injury significantly decreased the diameter of muscle fibers, but HBOT significantly increased the mean fiber cross-sectional area as compared with sham treatment after burn (95% CI: 153.0-986.8, p <0.05). According to picrosirius red staining and Masson's trichrome staining, HBOT mitigated the extent of fibrotic area after burn (Figure 2). Furthermore, the immunofluorescence in the gastrocnemius muscle showed that the expression of the E3 ubiquitin ligase MuRF-1, which is activated during muscle atrophy, was significantly decreased in the HBOT group (Figure 3A). We also assessed the level of atrogin-1, a muscle-specific protein that plays a key role in muscle atrophy, through Western blotting. The atrogin-1 expression in the untreated burn group was significantly higher than that in the control group and significantly lower in the HBOT group (95% CI 0.94-1.71, *p* <0.001) (Figure 3B). In addition, HBOT reduced the overexpression of cleaved caspase-3 and cleaved caspase-9 in the gastrocnemius muscle post-burn (Figure 3C). These results indicate that HBOT suppressed burn-induced muscle atrophy.

**Figure 4:**
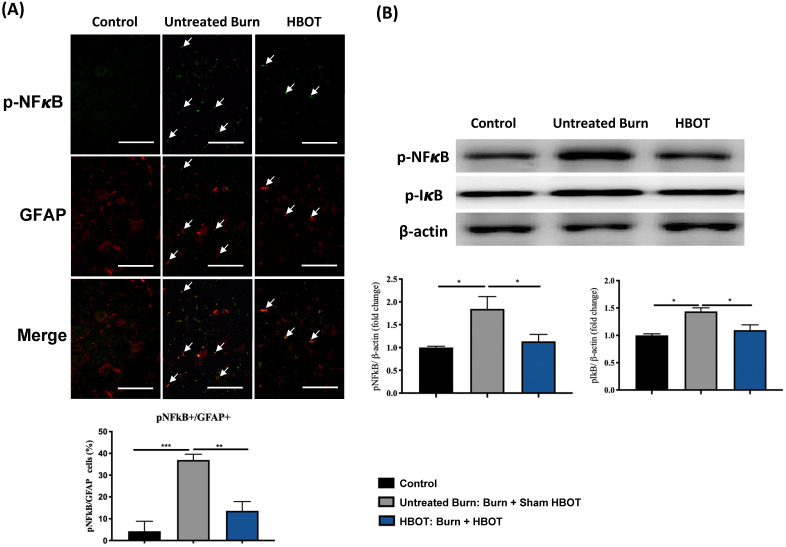
** HBOT inhibits burn-induced neuroinflammation in the ventral horn of spinal cord. (A)** Representative immunofluorescence images of GFAP (red) and NFkB (green) is the ventral horn of spinal cord. HBOT decreases NFkB-mediated astrocyte activation post-burn. (Scale bars, 50 µm). **(B)** Western blot analysis of p-NFkB and p-IkB in the ventral horn of spinal cord. HBOT attenuated the increase of p-NFkB and p-IkB following burn. *p < 0.05, **p < 0.01, ***p < 0.001 compared with indicated group.

**Figure 5:**
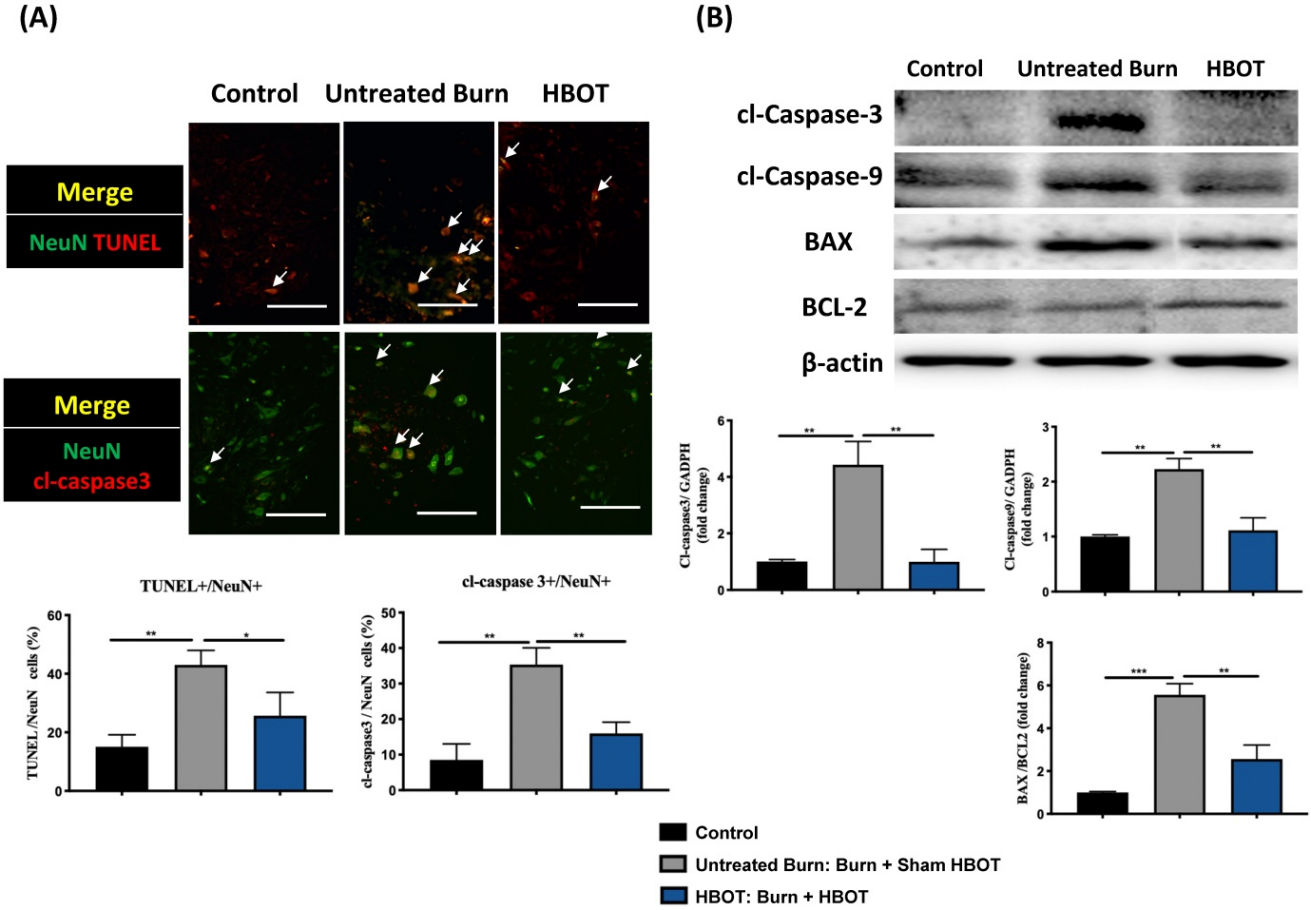
** HBOT attenuates burn-induced neuronal apoptosis in the ventral horn of spinal cord. (A)** Merged images of TUNEL assay (red) and NeuN (green). Decreased TUNEL-positive cells (double stained, arrows) in HBOT group. **(B)** Images of cleaved(cl)-caspase-3 (red)/NeuN (green) double immunofluorescence shows that HBOT decreases the number of caspase-3 positive neuron post-burn (double stained, arrows) (Scale bars, 100 µm). **(C)** Western blot analysis of cl-caspase-3, cl-caspase-9, BAX and BCL-2. A decrease of caspase cascades and BAX/BCL-2 ratio in HBOT group. *p < 0.05, **p < 0.01, ***p < 0.001 compared with indicated group.

**Figure 6:**
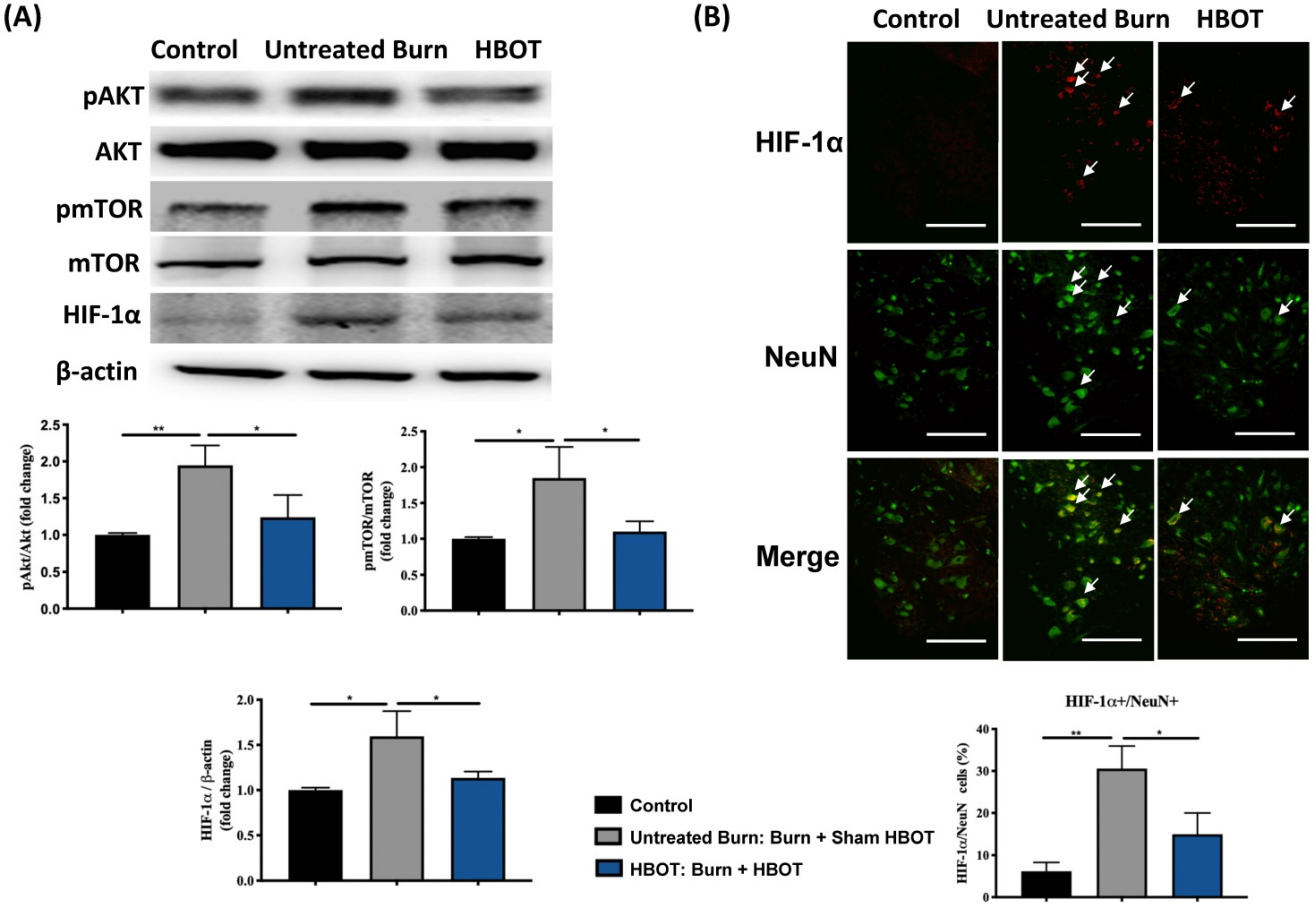
** HBOT attenuates burn-induced neuronal apoptosis by modulating AKT/mTOR/ HIF-1α. (A)** Western blot analysis of AKT, mTOR and HIF-1α. HBOT decreases the expression of AKT, mTOR and HIF-1α post-burn. **(B)** Immunofluorescence of HIF-1α (red) and NeuN (green). Images of HIF-1α (red)/NeuN (green) double immunofluorescence shows that HBOT decreases the number of HIF-1α positive neuron post-burn (double stained, arrows). (Scale bars, 100 µm). *p < 0.05, **p < 0.01 compared with indicated group.

In the ventral horn, the anti-inflammatory and anti-apoptotic effects of HBOT were investigated. HBOT decreased NFκB-mediated astrocyte activation post-burn, as determined through immunofluorescence (Figure 4A) and attenuated the overexpression of p-NF-κB and p-IκB, as determined through Western blotting (Figure 4B). Double-label staining of TUNEL with NeuN was performed to colocalize neuronal apoptosis; compared with the control, the TUNEL and NeuN-positive cells was more abundant in the untreated burn group, but apoptotic neuronal cells were less in the HBOT group (Figure 5A). Merged images revealed more cleaved capase-3 immunostaining in the NeuN-positive cells in the untreated burn group, but HBOT decreased the number of caspase-3-positive neurons post-burn. Furthermore, HBOT reduced the expression of cleaved caspase-3, cleaved caspase-9, and BAX (apoptosis regulator)/BCL-2 (anti-apoptotic protein) post-burn through Western blotting (Figure 5B). These results suggest that HBOT can reduce neuroinflammation and apoptosis in the ventral horn. We previously reported that the AKT/mTOR pathway was engaged in burn-induced motor neuron apoptosis 42. HIF-1α is a crucial regulator involved in tissue inflammation and cellular apoptosis. Western blotting showed that the expression of p-AKT/AKT, p-mTOR/mTOR, and HIF-1α was elevated post-burn, and clearly decreased after HBOT (Figure 6A). Double immunofluorescence also revealed that the HIF-1α- and NeuN-positive labeling cells increased in the untreated burn group and decreased in the HBOT group (Figure 6B).

## Discussion

In the present study, we investigated the therapeutic effect of delayed HBOT (2.5 ATA, 90 min/day), which was started at 5 weeks after burn injury for 14 treatments, and the therapeutic mechanism underlying the chronic phase of burn-induced neuromuscular damage after HBOT. HBOT reduced the gait disturbance of the injured limb as well as gastrocnemius muscle atrophy after burn injury in rats. Inflammation and apoptosis in the ventral horn contributing to denervated muscle atrophy were key pathogenic processes in the burn model. HBOT attenuated burn-induced neuroinflammation and neuronal apoptosis in the ventral horn of the spinal cord. Our findings also suggest that the therapeutic effect of HBOT involves the HIF-1α pathway.

HBOT has been reported to reduce tissue hypoxia, inflammatory response, and neovascularization. However, evidence of the benefits of HBOT in burn care remains insufficient 43-45. In addition, the optimal dosage and timing of HBOT are uncertain for burn care. Experimental and clinical studies suggest the use of HBOT in burn wound healing 46, 47. Adjunctive HBOT has been suggested to reduce postburn bacterial translocation and sepsis 48, 49. Furthermore, data increasingly support HBOT as a part of a neuroprotective strategy in stroke 50-52, neurodegenerative diseases 53-55 and traumatic brain injury 56 to improve functional recovery. In this study, we found a benefit of delayed HBOT (administered at days 28-41 after burn injury) for reducing long-term neuromuscular complications post-burn.

The effects of HBOT depend on dosage, pressure, duration, frequency, and the cumulative number of treatments. Our previous burn study in rats showed that 2-weeks HBOT sustained a protective effect longer than 1-week HBOT 38. In the present study, 14 sessions of HBOT at 2.5ATA (90 mins, once daily) attenuated cell apoptosis and inflammatory response in the ventral horn. Several studies have proposed that HBOT downregulates NF-κB signaling to attenuate the inflammatory reaction after spinal cord injury 57-59. In our experimental burn model, HBOT reduced the activation of p-NF-κB and p-IκB in ventral horn after burn injury. NF-κB plays a critical role in the control of cell division and apoptosis. The activation of NF-κB in microglia in response to injury promotes neuronal degeneration 60. Furthermore, an increase in the ratio of BAX to BCL-2 and the activation of caspase-3 and caspase-9 were found in the ventral horn after burn injury. HBOT markedly reduced the number of apoptotic neuronal cells post-burn and decreased the expression of BAX/BCL-2, caspase-3 and caspase-9.

In addition to denervated muscle atrophy after burn, our results showed that HBOT decreased the expression of caspases, and reduced the upregulation of E3-ubiquitin ligases in gastrocnemius tissue. HBOT promotes the recovery of induced muscle injury 61. Increased myogenesis 11, modulated efficiency of skeletal muscle mitochondria 62, and enhanced differentiation of satellite cells 63 have been reported to facilitate injured muscle recovery with HBOT. In this study, HBOT attenuated the expression of caspase-3 and caspase-9 in gastrocnemius muscle, indicating that HBOT protects muscle tissue from apoptosis. Ubiquitin-proteasome proteolytic pathway activation also plays an important role in burn-induced skeletal muscle wasting by accelerating the breakdown of myofibrillar proteins 64-66. Atrogin-1 and MuRF-1 are two muscle-specific E3 ubiquitin ligases that are excellent markers of muscle atrophy 67. HBOT decreased the expression atrogin-1 post-burn in gastrocnemius tissue by Western blotting analysis. Immunofluorescence also revealed that HBOT significantly reduced the number of MuRF-1-positive cells after burn injury.

The role of HIF-1α in HBOT attenuating burn-induced motor neuron death remains unknown. Previous reports have suggested that HBOT promotes neurogenesis by reducing the expression of HIF-1α 68, 69. In rat model of spinal cord injury, the protein production of HIF-1α was elevated after injury, but HBOT reduced the expression of HIF-1α 70. HIFs play important roles in physiological and pathological conditions, involving survival, the cell-cycle, and metabolism 71, 72. They are activated by hypoxic stress; however, it also frequently has been found in normoxic conditions 73, 74. Stiehl *et al.* hypothesized that HIF-1α was activated in normoxia by peptides such as insulin and interleukin‐1β through the phosphatidylinositol 3‐kinase pathway 74. A review paper also indicated possible molecular crosstalk between HIFs and NF‐κB for a variety of medical conditions 75 because HIFs are connected to inflammation and amplify NF-κB. Our results showed HBOT suppresses the activation of NF-κB, IκB and HIF-1α in the ventral horn to prevent neuronal apoptosis after burn injury. In addition, the AKT/mTOR pathway has been suggested to play a key role in the regulation of the cell cycle 76-78, and inhibiting AKT/mTOR signaling protected neurons from apoptosis 79. Moreover, AKT pathway is the major upstream mediator of HIF-1α activation regardless of oxygen concentrations 80, 81. Our previous study indicated that burn induced programmed cell death in the ventral horn through AKT/mTOR pathway 82. In this study, HBOT reduced the expression of p-AKT and p-mTOR and HIF-1α in the ventral horn in a rat model of burn injury. We suggested that HBOT attenuates motor neuron apoptosis by modulating AKT/mTOR/HIF-1α signaling pathway and inactivating apoptosis-associated proteins.

HBOT has great potential in the treatment of neuronal injuries 83-85. However, there are some limitations of our study. First, the optimal timing and total number treatments of HBOT require further researches to clarify the effectiveness of HBOT. Second, we chose to delay HBOT until burn wound healing; however, the efficacy of early HBOT was not evaluated in this model. Third, the role of HIF-1α in the therapeutic effect of HBOT in our burn model remains to be fully elucidated. Fourth, follow-up studies are needed to investigate the effectiveness HBOT in the long term. Fifth, whether HBOT have the synchronous effect on motor neuron and muscle repair needs further research to clarify.

## Conclusion

Our study suggests that HBOT mitigates burn-induced neuronal apoptosis in ventral horn post-burn by modulating HIF-1α signaling. HBOT further attenuated denervated gastrocnemius muscle atrophy and fibrotic changes after burn injury.

## Abbreviations

HIF-1α: hypoxia-inducible factor-1α
HBOT: Hyperbaric oxygen therapy
CNS: central nervous system
H&E stain: hematoxylin and eosin stain
MuRF-1: muscle ring finger-1
TUNEL assay: terminal deoxynucleotidyl transferase dUTP nick-end labeling assay
NeuN: Neuron-specific nuclear protein
GFAP: glial fibrillary acidic protein
cl-caspase-3: cleaved-caspase-3
cl-caspase-9: cleaved-caspase-9
BAX: BCL-2-associated X
BCL-2: B-cell lymphoma 2
mTOR: mammalian target of rapamycin

## References

[1] Hart DW, Wolf SE, Mlcak R (2000). Persistence of muscle catabolism after severe burn. Surgery.

[2] Wu X, Baer LA, Wolf SE (2010). The impact of muscle disuse on muscle atrophy in severely burned rats. J Surg Res.

[3] Rinkinen J, Hwang CD, Agarwal S (2015). The Systemic Effect of Burn Injury and Trauma on Muscle and Bone Mass and Composition. Plast Reconstr Surg.

[4] Polychronopoulou E, Herndon DN, Porter C (2018). The Long-Term Impact of Severe Burn Trauma on Musculoskeletal Health. J Burn Care Res.

[5] Pereira C, Murphy K, Jeschke M (2005). Post burn muscle wasting and the effects of treatments. Int J Biochem Cell Biol.

[6] Clark A, Imran J, Madni T (2017). Nutrition and metabolism in burn patients. Burns Trauma.

[7] Wu SH, Huang SH, Cheng KI (2015). Third-Degree Hindpaw Burn Injury Induced Apoptosis of Lumbar Spinal Cord Ventral Horn Motor Neurons and Sciatic Nerve and Muscle Atrophy in Rats. Biomed Research International.

[8] Ma L, Zhou Y, Khan MAS (2019). Burn-Induced Microglia Activation is Associated With Motor Neuron Degeneration and Muscle Wasting in Mice. Shock.

[9] Tibbles PM, Edelsberg JS (1996). Hyperbaric-oxygen therapy. N Engl J Med.

[10] Novak S, Drenjancevic I, Vukovic R (2016). Anti-Inflammatory Effects of Hyperbaric Oxygenation during DSS-Induced Colitis in BALB/c Mice Include Changes in Gene Expression of HIF-1alpha, Proinflammatory Cytokines, and Antioxidative Enzymes. Mediators Inflamm.

[11] Oyaizu T, Enomoto M, Yamamoto N (2018). Hyperbaric oxygen reduces inflammation, oxygenates injured muscle, and regenerates skeletal muscle via macrophage and satellite cell activation. Sci Rep.

[12] Chen X, Li Y, Chen W (2016). Protective Effect of Hyperbaric Oxygen on Cognitive Impairment Induced by D-Galactose in Mice. Neurochem Res.

[13] Shwe T, Bo-Htay C, Ongnok B (2021). Hyperbaric oxygen therapy restores cognitive function and hippocampal pathologies in both aging and aging-obese rats. Mech Ageing Dev.

[14] Xu Y; Wang Q; Qu Z (2019). Protective Effect of Hyperbaric Oxygen Therapy on Cognitive Function in Patients with Vascular Dementia. Cell Transplant.

[15] Mu J, Ostrowski RP, Soejima Y (2013). Delayed hyperbaric oxygen therapy induces cell proliferation through stabilization of cAMP responsive element binding protein in the rat model of MCAo-induced ischemic brain injury. Neurobiol Dis.

[16] Lim SW, Sung KC, Shiue YL (2017). Hyperbaric Oxygen Effects on Depression-Like Behavior and Neuroinflammation in Traumatic Brain Injury Rats. World Neurosurgery.

[17] Zhang Y, Yang Y, Tang H (2014). Hyperbaric oxygen therapy ameliorates local brain metabolism, brain edema and inflammatory response in a blast-induced traumatic brain injury model in rabbits. Neurochem Res.

[18] Cheng O, Ostrowski RP, Wu B (2011). Cyclooxygenase-2 mediates hyperbaric oxygen preconditioning in the rat model of transient global cerebral ischemia. Stroke.

[19] Pan JY, Cai RX, Chen Y (2018). Analysis the effect of hyperbaric oxygen preconditioning on neuronal apoptosis, Ca2+ concentration and caspases expression after spinal cord injury in rats. Eur Rev Med Pharmacol Sci.

[20] Zhou Y, Su P, Pan Z (2019). Combination Therapy With Hyperbaric Oxygen and Erythropoietin Inhibits Neuronal Apoptosis and Improves Recovery in Rats With Spinal Cord Injury. Phys Ther.

[21] Dave KR, Prado R, Busto R (2003). Hyperbaric oxygen therapy protects against mitochondrial dysfunction and delays onset of motor neuron disease in Wobbler mice. Neuroscience.

[22] Zhang Q, Chang Q, Cox RA (2008). Hyperbaric oxygen attenuates apoptosis and decreases inflammation in an ischemic wound model. J Invest Dermatol.

[23] Nakazawa H, Ikeda K, Shinozaki S (2017). Burn-induced muscle metabolic derangements and mitochondrial dysfunction are associated with activation of HIF-1alpha and mTORC1: Role of protein farnesylation. Sci Rep.

[24] Li A, Sun X, Ni Y (2013). HIF-1alpha involves in neuronal apoptosis after traumatic brain injury in adult rats. J Mol Neurosci.

[25] Fang Y, Lu J, Wang X (2020). HIF-1alpha Mediates TRAIL-Induced Neuronal Apoptosis via Regulating DcR1 Expression Following Traumatic Brain Injury. Front Cell Neurosci.

[26] Sun JJ, Zhang XY, Qin XD (2019). MiRNA-210 induces the apoptosis of neuronal cells of rats with cerebral ischemia through activating HIF-1alpha-VEGF pathway. Eur Rev Med Pharmacol Sci.

[27] Yang XS, Yi TL, Zhang S (2017). Hypoxia-inducible factor-1 alpha is involved in RIP-induced necroptosis caused by *in vitro* and *in vivo* ischemic brain injury. Sci Rep.

[28] Chen H, Zheng J, Ma J (2019). Vanillin ameliorates changes in HIF-1alpha expression and neuronal apoptosis in a rat model of spinal cord injury. Restor Neurol Neurosci.

[29] Imtiyaz HZ, Simon MC (2010). Hypoxia-inducible factors as essential regulators of inflammation. Curr Top Microbiol Immunol.

[30] Shay JE, Celeste Simon M (2012). Hypoxia-inducible factors: crosstalk between inflammation and metabolism. Semin Cell Dev Biol.

[31] Lin N, Simon MC (2016). Hypoxia-inducible factors: key regulators of myeloid cells during inflammation. J Clin Invest.

[32] Koh HS, Chang CY, Jeon SB (2015). The HIF-1/glial TIM-3 axis controls inflammation-associated brain damage under hypoxia. Nat Commun.

[33] Guo K, Yao X, Wu W (2020). HIF-1alpha/SDF-1/CXCR4 axis reduces neuronal apoptosis via enhancing the bone marrow-derived mesenchymal stromal cell migration in rats with traumatic brain injury. Exp Mol Pathol.

[34] Abdel-Latif RG, Rifaai RA, Amin EF (2020). Empagliflozin alleviates neuronal apoptosis induced by cerebral ischemia/reperfusion injury through HIF-1alpha/VEGF signaling pathway. Arch Pharm Res.

[35] Liu J, Narasimhan P, Yu F (2005). Neuroprotection by hypoxic preconditioning involves oxidative stress-mediated expression of hypoxia-inducible factor and erythropoietin. Stroke.

[36] Li J, Tao T, Xu J (2020). HIF1alpha attenuates neuronal apoptosis by upregulating EPO expression following cerebral ischemiareperfusion injury in a rat MCAO model. Int J Mol Med.

[37] Mering S, Kaliste-Korhonen E, Nevalainen T (2001). Estimates of appropriate number of rats: interaction with housing environment. Lab Anim.

[38] Wu ZS, Wu SH, Lee SS (2019). Dose-Dependent Effect of Hyperbaric Oxygen Treatment on Burn-Induced Neuropathic Pain in Rats. Int J Mol Sci.

[39] Tezcan O, Karahan O, Alan M (2017). Hyperbaric Oxygen Preconditioning Provides Preliminary Protection Against Doxorubicin Cardiotoxicity. Acta Cardiol Sin.

[40] Chen MJ, Chen TY, Cheng YM (2012). The effect of postoperative hyperbaric oxygen treatment on intra-abdominal adhesions in rats. Int J Mol Sci.

[41] Na HS, Kwon JY, Lee SY (2021). Metformin Attenuates Monosodium-Iodoacetate-Induced Osteoarthritis via Regulation of Pain Mediators and the Autophagy-Lysosomal Pathway. Cells.

[42] Wu SH, Lu IC, Lee SS (2018). Erythropoietin attenuates motor neuron programmed cell death in a burn animal model. Plos One.

[43] Weitgasser L, Ihra G, Schafer B (2021). Update on hyperbaric oxygen therapy in burn treatment. Wien Klin Wochenschr.

[44] Edwards M, Singh M, Selesny S (2021). Hyperbaric Treatment Of Thermal Burns. In StatPearls, Treasure Island (FL).

[45] Smolle C, Lindenmann J, Kamolz L (2021). The History and Development of Hyperbaric Oxygenation (HBO) in Thermal Burn Injury. Medicina (Kaunas).

[46] Oley MH, Oley MC, Aling DMR (2021). Effects of hyperbaric oxygen therapy on the healing of thermal burns and its relationship with ICAM-1: A case-control study. Ann Med Surg (Lond).

[47] Hatibie MJ, Islam AA, Hatta M (2019). Hyperbaric Oxygen Therapy for Second-Degree Burn Healing: An Experimental Study in Rabbits. Adv Skin Wound Care.

[48] Chiang IH, Chen SG, Huang KL (2017). Adjunctive hyperbaric oxygen therapy in severe burns: Experience in Taiwan Formosa Water Park dust explosion disaster. Burns.

[49] Akin ML, Gulluoglu BM, Erenoglu C (2002). Hyperbaric oxygen prevents bacterial translocation in thermally injured rats. J Invest Surg.

[50] Cozene B, Sadanandan N, Gonzales-Portillo B (2020). An Extra Breath of Fresh Air: Hyperbaric Oxygenation as a Stroke Therapeutic. Biomolecules.

[51] Schiavo S, Richardson D, Santa Mina D (2020). Hyperbaric oxygen and focused rehabilitation program: a feasibility study in improving upper limb motor function after stroke. Appl Physiol Nutr Metab.

[52] Hadanny A, Rittblat M, Bitterman M (2020). Hyperbaric oxygen therapy improves neurocognitive functions of post-stroke patients - a retrospective analysis. Restor Neurol Neurosci.

[53] Shapira R, Solomon B, Efrati S (2018). Hyperbaric oxygen therapy ameliorates pathophysiology of 3xTg-AD mouse model by attenuating neuroinflammation. Neurobiol Aging.

[54] BenAri O, Efrati S, Sano M (2020). A double-blind placebo-controlled clinical trial testing the effect of hyperbaric oxygen therapy on brain and cognitive outcomes of mildly cognitively impaired elderly with type 2 diabetes: Study design. Alzheimers Dement (N Y).

[55] Harch PG, Fogarty EF Hyperbaric oxygen therapy for Alzheimer's dementia with positron emission tomography imaging: a case report. Med Gas Res. 2018: 8: 181-4.

[56] He H, Li X, He Y (2019). Hyperbaric oxygen therapy attenuates neuronal apoptosis induced by traumatic brain injury via Akt/GSK3beta/beta-catenin pathway. Neuropsychiatr Dis Treat.

[57] Sun L, Zhao L, Li P (2019). Effect of hyperbaric oxygen therapy on HMGB1/NF-kappaB expression and prognosis of acute spinal cord injury: A randomized clinical trial. Neurosci Lett.

[58] Kang N, Hai Y, Yang J (2015). Hyperbaric oxygen intervention reduces secondary spinal cord injury in rats via regulation of HMGB1/TLR4/NF-kappaB signaling pathway. Int J Clin Exp Pathol.

[59] Tan J, Zhang F, Liang F (2014). Protective effects of hyperbaric oxygen treatment against spinal cord injury in rats via toll-like receptor 2/nuclear factor-kappaB signaling. Int J Clin Exp Pathol.

[60] Mattson MP, Camandola S (2001). NF-kappaB in neuronal plasticity and neurodegenerative disorders. J Clin Invest.

[61] Cervaens Costa Maia M, Camacho OF, Pinto Marques AF (2013). Hyperbaric oxygen therapy treatment for the recovery of muscle injury induced in rats. Diving Hyperb Med.

[62] Cervaens M, Lumini-Oliveira J, Ascensao A (2018). The influence of hyperbaric environment on the skeletal muscle mitochondrial energetic of rats after induced muscle contusion. Undersea Hyperb Med.

[63] Horie M, Enomoto M, Shimoda M (2014). Enhancement of satellite cell differentiation and functional recovery in injured skeletal muscle by hyperbaric oxygen treatment. J Appl Physiol (1985).

[64] Chai J, Wu Y, Sheng Z (2002). The relationship between skeletal muscle proteolysis and ubiquitin-proteasome proteolytic pathway in burned rats. Burns.

[65] Ma L, Chu W, Chai J (2017). ER stress and subsequent activated calpain play a pivotal role in skeletal muscle wasting after severe burn injury. PLoS One.

[66] Attaix D, Ventadour S, Codran A (2005). The ubiquitin-proteasome system and skeletal muscle wasting. Essays Biochem.

[67] Bodine SC, Baehr LM (2014). Skeletal muscle atrophy and the E3 ubiquitin ligases MuRF1 and MAFbx/atrogin-1. Am J Physiol Endocrinol Metab.

[68] Mu J, Krafft PR, Zhang JH (2011). Hyperbaric oxygen therapy promotes neurogenesis: where do we stand?. Med Gas Res.

[69] Hu Q, Liang X, Chen D (2014). Delayed hyperbaric oxygen therapy promotes neurogenesis through reactive oxygen species/hypoxia-inducible factor-1alpha/beta-catenin pathway in middle cerebral artery occlusion rats. Stroke.

[70] Zhou Y, Liu XH, Qu SD (2013). Hyperbaric oxygen intervention on expression of hypoxia-inducible factor-1alpha and vascular endothelial growth factor in spinal cord injury models in rats. Chin Med J (Engl).

[72] Karshovska E, Wei Y, Subramanian P (2020). HIF-1alpha (Hypoxia-Inducible Factor-1alpha) Promotes Macrophage Necroptosis by Regulating miR-210 and miR-383. Arterioscler Thromb Vasc Biol.

[73] Dery MA, Michaud MD, Richard DE (2005). Hypoxia-inducible factor 1: regulation by hypoxic and non-hypoxic activators. Int J Biochem Cell Biol.

[74] Stiehl DP, Jelkmann W, Wenger RH (2002). Normoxic induction of the hypoxia-inducible factor 1alpha by insulin and interleukin-1beta involves the phosphatidylinositol 3-kinase pathway. FEBS Lett.

[75] D'Ignazio L, Bandarra D, Rocha S (2016). NF-kappaB and HIF crosstalk in immune responses. FEBS J.

[76] Feng H, Cheng X, Kuang J (2018). Apatinib-induced protective autophagy and apoptosis through the AKT-mTOR pathway in anaplastic thyroid cancer. Cell Death Dis.

[77] Liu Q, Qiu J, Liang M (2014). Akt and mTOR mediate programmed necrosis in neurons. Cell Death & Disease.

[78] Yu JS (2016). Cui W. Proliferation, survival and metabolism: the role of PI3K/AKT/mTOR signalling in pluripotency and cell fate determination. Development.

[79] Xu XK, Wang SY, Chen Y (2018). Fangjing decoction relieves febrile seizures-induced hippocampal neuron apoptosis in rats via regulating the Akt/mTOR pathway. Biosci Rep.

[80] Agani F, Jiang BH (2013). Oxygen-independent regulation of HIF-1: novel involvement of PI3K/AKT/mTOR pathway in cancer. Curr Cancer Drug Targets.

[81] Iommarini L, Porcelli AM, Gasparre G (2017). Non-Canonical Mechanisms Regulating Hypoxia-Inducible Factor 1 Alpha in Cancer. Front Oncol.

[82] Wu SH, Lu IC, Lee SS (2018). Erythropoietin attenuates motor neuron programmed cell death in a burn animal model. PLoS One.

[83] Shams Z, Khalatbary AR, Ahmadvand H (2017). Neuroprotective effects of hyperbaric oxygen (HBO) therapy on neuronal death induced by sciatic nerve transection in rat. BMC Neurol.

[84] Zhao B, Pan Y, Wang Z (2017). Hyperbaric Oxygen Pretreatment Improves Cognition and Reduces Hippocampal Damage Via p38 Mitogen-Activated Protein Kinase in a Rat Model. Yonsei Med J.

[85] Yang L, Hei MY, Dai JJ (2016). Effect of hyperbaric oxygenation on mitochondrial function of neuronal cells in the cortex of neonatal rats after hypoxic-ischemic brain damage. Braz J Med Biol Res.

